# New Medical Books

**Published:** 1903-10

**Authors:** 


NEW MEDICAL BOOKS.

We have just received the 1903 Autumn Announcement of Medical Publications, issued by Lea Bros. & Co. Short reviews of many excellent medical books are contained therein. The catalogue will be sent upon request to the address of any physician in need of books.

